# CTRP7 Is a Biomarker Related to Insulin Resistance and Oxidative Stress: Cross-Sectional and Intervention Studies In *Vivo* and In *Vitro*

**DOI:** 10.1155/2022/6877609

**Published:** 2022-03-23

**Authors:** Kejia Li, Zerong Liang, Weiwei Xu, Han Dai, Mingyuan Tian, Ling Li, Hua Liu, Chen Chen, Dongfang Liu, Hongting Zheng, Zhiming Zhu, Gangyi Yang, Mengliu Yang

**Affiliations:** ^1^Department of Endocrinology, The Second Affiliated Hospital, Chongqing Medical University, 400010 Chongqing, China; ^2^Key Laboratory of Diagnostic Medicine (Ministry of Education) and Department of Clinical Biochemistry, College of Laboratory Medicine, Chongqing Medical University, China; ^3^Department of Pediatrics, University of Mississippi Medical Center, 2500 North State Street, Jackson, Mississippi, MS 39216-4505, USA; ^4^Endocrinology, SBMS, Faculty of Medicine, University of Queensland, Brisbane 4072, Australia; ^5^Department of Endocrinology, Xinqiao Hospital, Army Medical University, Chongqing 400014, China; ^6^Department of Hypertension and Endocrinology, Daping Hospital, Army Medical University, Chongqing Institute of Hypertension, Chongqing 400010, China

## Abstract

**Objective:**

CTRP7 is a cytokine that is known to be associated with obesity. However, its relationship with insulin resistance (IR) and metabolic diseases remains unknown. The aim of this study is to investigate the relationship between CTRP7 and IR under *in vivo* and *in vitro* conditions.

**Methods:**

CTRP7 expression in mice and hepatocytes was determined using RT-qPCR and western blotting. Circulating CTRP7 concentrations were measured with an ELISA kit. EHC, OGTT, lipid-infusion, physical activity, and cold-stimulation experiments were performed in humans and mice. SOD, GSH, and MDA were measured by commercial kits. ROS levels were detected using dichlorofluorescein diacetate.

**Results:**

The expression levels of CTRP7 protein in the liver and fat of ob/ob and db/db mice were higher than that of WT mice. Individuals with IGT, T2DM, and obesity had higher circulating CTRP7 levels. CTRP7 levels were associated with HOMA-IR, obesity, and other metabolic parameters. During OGTT, serum CTRP7 levels gradually decreased, while CTRP7 levels significantly increased during EHC in response to hyperinsulinemia in healthy individuals without IR. In addition, lipid infusion-induced IR further increased serum CTRP7 levels in healthy adults. Physical activity increased serum CTRP7 levels in healthy individuals and CTRP7 protein expression in iWAT and skeletal muscle in mice. Under *in vitro* conditions, the expression of the CTRP7 protein was inhibited in a glucose concentration-dependent manner but was promoted by FFAs and insulin stimulation in hepatocytes. Furthermore, CTRP7 overexpression facilitated oxidative stress and suppressed the phosphorylation of insulin signaling molecules in hepatocytes.

**Conclusions:**

Our evidence shows that CTRP7 could be a useful biomarker and potential treatment target in IR and metabolic disorders.

## 1. Introduction

Adipose tissue, which is the largest endocrine organ in the body, secretes a large number of bioactive peptide hormones into blood circulation that are collectively referred to as adipokines [1]. Adipokines play a key role in regulating insulin signaling and metabolism. In recent years, a conserved family of adipokines with a C-terminal complement factor (C1q) globular domain which is similar to that found in insulin sensitizers, adiponectin (ADIPOQ), has been identified as part of the C1q/tumor necrosis factor-related protein (CTRP) family [2]. These proteins have four different domains that are similar to those of ADIPOQ: an N-terminal signal peptide, a short variable region, a collagen domain with different length Gly-X-Y repeats, and a C1q domain in the C-terminal [3]. The CTRP family was derived from a precursor molecule of the innate immune system, and in the course of evolution, this protein family has been expanding [4]. Currently, seven known members of the CTRP family are designated CTRP-1 to CTRP-7 [2, 5]. Some CTRP proteins secreted by adipose tissue, such as CTRP1, CTRP3, CTRP5, and CTRP7, are involved in adipocyte differentiation, apoptosis, autoimmunity, energy metabolism, and insulin signaling [6–9].

The circulating levels of CTRPs may vary according to genetic background, gender, and metabolic status [10]. Although the physiological function of some members of the CTRP family has been well described, CTRP7 is not entirely clear and must be further studied.

Recently, a small-scale population study showed that circulating CTRP7 levels are elevated in individuals with obesity and are related to blood glucose level, body mass index, and insulin resistance (IR) [11]. Accordingly, animal studies have also shown that the expression of CTRP7 mRNA in adipose tissue was increased in diabetic mice [9]; further, CTRP7 deficiency could improve IR and impaired glucose tolerance (IGT) [11]. Another study reported that CTRP7 expression was upregulated in the skeletal muscle of aged rats and further increased in response to caloric restriction [12]. These data indicate that CTRP7 plays a role in whole-body energy metabolism and IR and can be secreted by fat and muscle tissue. However, the previous studies which reported these findings were observational cross-sectional population studies conducted on small samples. Therefore, there is a lack of large-scale intervention studies and in-depth research under *in vivo* and *in vitro* conditions.

In the present study, we investigated circulating CTRP7 levels in individuals who were newly diagnosed with type 2 diabetes mellitus (T2DM) and prediabetes (IGT) and performed various intervention experiments to observe the effects of hyperglycemia, hyperinsulinemia, free fatty acid- (FFA-) induced IR, physical activity, and cold exposure on serum CTRP7 levels. Additionally, we measured the level of CTRP7 protein in insulin target tissues of mice that were fed a normal chow diet (ND) or high-fat diet (HFD) and mice with diabetes (db/db and ob/ob mice) and also performed a series of intervention studies.

## 2. Material and Methods

### 2.1. Human Study

#### 2.1.1. A Cross-Sectional Study in a Population

624 individuals, including 288 T2DM, 168 IGT individuals, and 168 normal individuals (NGT) were recruited from outpatients attending the Department of Endocrinology at the Second Affiliated Hospital, Chongqing Medical University, as well as from advertisements or routine medical examinations. The diagnosis of T2DM and IGT was based on the diagnostic criteria of the World Health Organization (WHO) in 1998 [13]. All patients with IGT and T2DM were newly diagnosed and did not receive any hypoglycemic drugs or lifestyle intervention, other inclusion criteria included age 40-70 years, BMI 20-40 kg/m^2^. Patients with type 1 diabetes mellitus (T1DM), hypertension, liver cirrhosis, liver and kidney failure, congestive heart failure, and the complications of diabetes or other chronic diseases were excluded from the study. In addition, the control individuals did not take any drugs that affect glucose metabolism and insulin sensitivity. All participants signed informed consent. The study was approved by the Human Research Ethics Committee of Chongqing Medical University, following the Declaration of Helsinki, and registered at the Chinese Clinical Trial Registry (CHICTR-OCS-13003185).

#### 2.1.2. Anthropometric and Biochemical Measurements

BMI was calculated based on body weight divided by the square of height. Waist circumference (WC) and hip circumference (HC) were measured, and waist-hip ratio (WHR) was calculated by WC/HC. Blood pressure was measured after resting for at least 15 minutes. Body fat percentage was measured by the bio-impedance method (bia-101; RJL system, Shenzhen, China). HOMA-IR was calculated as follows: HOMA − IR = fasting insulin (FIns, mU/L) × fasting blood glucose (FBG, mmol/L)/22.5; HOMA-*β* was calculated as follows: HOMA − *β* = 20 × FIns/(FBG − 3.5) [14]. Glucose and HbA1c were determined by glucose oxidase and anion-exchange HPLC, respectively. Serum insulin was measured by chemiluminescence. FFAs and blood fat were measured as reported previously [15].

#### 2.1.3. Oral Glucose Tolerance Test (OGTT) and Euglycemic-Hyperinsulinemic Clamps (EHC)

OGTTs were performed in all study populations. At 7 a.m., all individuals restricted from food overnight were given glucose (75 g). Blood was taken at the designated time (0, 30, 60, 120 min) for measuring blood glucose, insulin, adiponectin, and CTRP7.

EHCs were performed in 30 healthy individuals (15 men and 15 women; age 26.7 ± 3.1 years; BMI 21.4 ± 2.1 kg/m^2^), as previously described [16]. Briefly, the experiment started at 8 : 00 a.m. after participants fasted overnight, and catheters were inserted into the left and right elbow anterior veins for collecting blood and infusing glucose and insulin. During the EHC, normal human insulin (1 mU/kg/min) was infused for 2 h, and 20% glucose was infused at a variable rate to maintain blood glucose at fasting levels. The EHC experiment lasted for 120 min, and blood glucose was detected every 15 min. Glucose disposal rate (GDR) was defined as the glucose infusion rate (GIR) in the stable phase of the EHC, which was related to body weight (M-value). Venous blood was collected at the indicated time (0, 80, 100, 110, and 120 min) and stored at -80°C until use.

#### 2.1.4. Lipid-Infused Study

To establish lipid-induced IR, 34 healthy participants (18 males and 16 females; age 25.4 ± 3.1 yr; BMI 21.2 ± 2.2 kg/m^2^) were performed a lipid-infusion combined with EHC. These participants received an infusion of 20% intralipid/heparin (0.4 U/kg/min) for 240 min (1.5 ml/min). 2 h after lipid-infusion, the EHC studies were started until the end of the experiment, as previously reported [15]. In the experiment, blood was collected at the indicated time and stored at –80°C.

#### 2.1.5. Physical Activity Experiment in Humans

Twenty-nine healthy participants (15 males and 14 females; age 24.8 ± 2.2 yr; BMI 21.3 ± 2.3 kg/m^2^) participated in the physical-activity study. Individuals who were taking antidiabetic drugs and had physical-activity contraindications, or who participated in the exercise for 20 min or more at least twice a week, were excluded from this study. After overnight fasting, the individuals arrived at the ward from 8 : 00 to 8 : 30 in the morning and participated in a 45-minutes treadmill experiment under the condition of 60% V_O2_ max (ParvoMedics Metabolic Measurement System, ParvoMedics). Blood was collected at four-time points, including baseline, 0 min, 60 min, and 120 min after exercise [15].

#### 2.1.6. Cold-Exposure Study in Humans

30 healthy individuals participated in the cold-exposure study (15 males and 15 females; age 24 ± 1.3 yr; BMI 21.8 ± 1.8 kg/m^2^). After overnight fasting, the individuals with light clothes were placed in a hospital bed with a water-circulated cooling blanket. Electromyography (EMG) was used for regulating temperature to avoid shaking. After 30 min of exposure to 27°C, a blood sample was drawn. Then, water temperature was cooled to 18°C. After that, the temperature was reduced by 2°C every 3 min to 12°C. Blood samples were taken after 5 min at 12°C for CTRP7 measurement [15].

#### 2.1.7. Determination of Serum CTRP7 and Adiponectin

Serum CTRP7 concentration was determined by an ELISA Kit according to the manufacturer's procedure (sk00396-09, Aviscerabio science Inc., USA). This ELISA kit has high sensitivity and specificity for the detection of human CTRP7 without obvious cross-reaction and interference. The intra- and interassay coefficients of variation (CV) were 4-6% and 8-10%, respectively. As previously reported [17], serum ADIPOQ concentrations were measured by an ELISA (sk00010-01, Aviscerabio science Inc., USA), with intra- and interassay CV of 4-8% and 8-12%, respectively.

### 2.2. Animal Study

Eight-week-old male C57BL/6 J (WT), db*/*db, and ob/ob mice were purchased from the Model Animal Research Center of Nanjing University. Mice were adaptively fed for 7 days and then fed with a normal diet (ND, 11% fat) for 4 weeks. To establish diet-induced obesity, eight-week-old male WT mice were fed an ND or HFD (60% kcal from fat; D12492, Research Diets, New Brunswick, NJ) for 12 weeks. For the exercise study, WT mice ran on a treadmill device for 60 min. Briefly, mice ran adaptively for 10 min, then the speed increased by 2 m/min until the maximum of 14 m/min, lasting for 60 min. Mice then were euthanized by isoflurane. Skeletal muscle and inguinal adipose tissue (iWAT) were collected and frozen immediately in liquid nitrogen. The animal experiments were approved by the animal studies committee of Chongqing Medical University. They were consistent with the National Institutes of Health Guide for the Care and Use of Laboratory Animals (NIH Publications No. 8023, revised 1978).

### 2.3. In Vitro Experiment

#### 2.3.1. Cell Culture and Treatments

HepG2 and Hepa1-6 cells (ATCC, Manassas, VA, USA) were cultured in DMEM as previously reported [15]. For insulin stimulation experiment, cells were cultured in a serum-free medium and subsequently stimulated with 10 nM of human insulin (Novo Nordisk, Denmark) or BSA for 2 h, 4 h, 8 h, 12 h, 24 h, and 48 h. To explore the effects of glucose on CTRP7 expression, cells were treated with glucose (44, 22, 11, and 5.5 mmol/L) for 24 h. For the fatty acids-stimulation experiment, cells were treated with 100 *μ*mol/L palmitate, oleate, or free fatty acid (FFAs) mixture (oleate/palmitate 2 : 1, Sigma- Aldrich, St. Louis, MO) or BSA as a control for 24 h. For investigating the role of CTRP7 on IR and oxidative stress, Hepa1-6 cells were transfected with a plasmid expressing CTRP7 (pLVX-*CTRP7*) (Tsingke Biotechnology, Beijing, China) or empty vector (pLVX-Puro) for 24 h, then exposed to 1% BSA or an FFAs (oleate: palmitate at 2 : 1) for another 24 h, as previously reported [18]. The plasmid sequences for CTRP7 were as follows: 5′-CGCAAATGGGC GGTAGGCGTG-3′ (forward) and 5′-AGCTACCCAGAAGAA AGACT-3′ (reverse). For insulin signal examination, the cells were stimulated with insulin or PBS for 20 minutes.

#### 2.3.2. Measurement of Oxidative Stress Markers

Antioxidant enzymes, including superoxide dismutase (SOD), glutathione (GSH), and malondialdehyde (MDA) as a biomarker of oxidative stress, were measured by their specific assay kits (Beyotime, Inc. Shanghai, China), according to the manufacturer's instructions.

To assess intracellular reactive oxygen species (ROS) formation, HepG2 cells treated with FFAs were incubated with dichloro-dihydro-fluorescein diacetate (DCFH-DA, 10 *μ*M) at 37°C for 60 min [19]. ROS fluorescence in the cells was recorded with a fluorescence microscope (Olympus Corporation, Tokyo, Japan), and intensity was analyzed using ImageJ software.

#### 2.3.3. mRNA and Protein Analysis

Real-time quantitative PCR (RT-qPCR) was performed as described previously using *β*-actin as an internal control gene [20]. The primer pairs used were listed in Supplementary Table 1. As described previously, protein expression was measured using western blotting [21]. Antibodies included anti-CTRP7 (Abcam, Cambridge, UK), anti-insulin receptor (InsR)/phospho-InsR^Tyr1150^, anti-AKT^ser473^/phospho-AKT, anti-FoxO1^ser256^/phosphor-FoxO1 (Cell Signaling Technology, Danvers, MA, USA), anti-GAPDH, and anti–*β*-actin (Santa Cruz Biotechnology, Dallas, TX, USA).

#### 2.3.4. Statistical Analysis

SPSS 25.0 version (Armonk, NY, USA) was used for statistical analysis. Kolmogorov-Smirnov test was performed for examining the distribution of the data. The variable of the normal distribution is expressed as mean ± SD or SE, and skewness variables were expressed as median (interquartile interval). The parameters of nonnormal distribution were transformed logarithmically before analysis. The differences between groups were determined by paired or unpaired student *t-*test, ANOVA, Kruskal-Wallis test, and Mann–Whitney *U* test. Spearman correlation coefficient and multiple linear regression were used to analyze the relationship between CTRP7 and other parameters. The binary logistic regression model was used to control the possible confounding variables and to assess the relationship between CTRP7 and T2DM and IGT. The receiver operating characteristics (ROC) curve was used to predict the cut-off of CTRP7 concentration in IGT and T2DM. In all statistical analysis, *p* < 0.05 was considered significant.

## 3. Results

### 3.1. CTRP7 Expression at Protein Levels in Obese and Diabetic Mice

**Figure 1:**
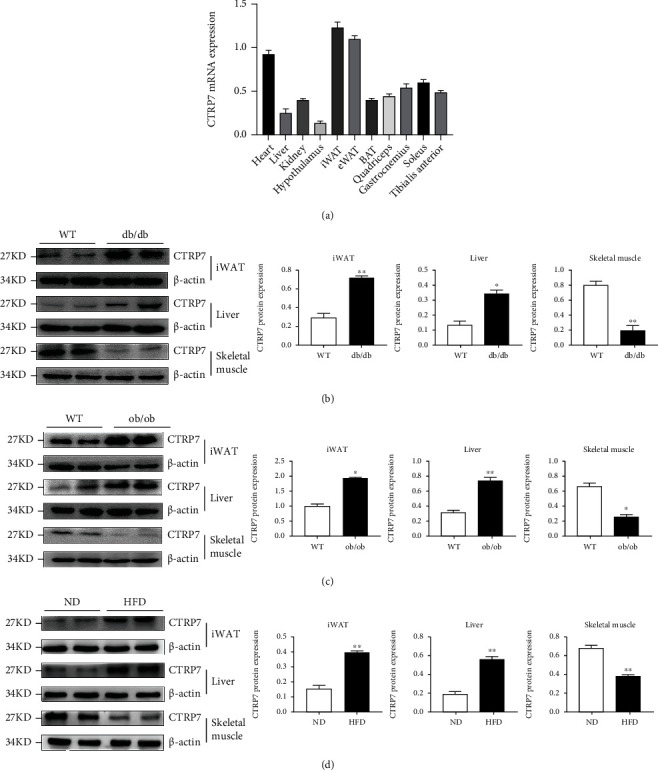
CTRP7 mRNA and protein expression in mice. (a) Tissue distribution of CTRP7 mRNA expression in C57BL/6 J (WT) mice. (b) CTRP7 protein expression in the liver, adipose tissue, and skeletal muscles of WT mice and db/db mice (*n* = 3). (c) CTRP7 protein expression in the liver, adipose tissue, and skeletal muscles of WT mice and ob/ob mice (*n* = 3). (d) CTRP7 protein expression in the liver, adipose tissue, and skeletal muscles of ND- or HFD-fed mice (*n* = 3). ND: normal diet; HFD: high-fat diet. Data are presented as means ± SEM. ∗*p* < 0.05 or ∗∗*p* < 0.01 vs. WT or ND.

We first engaged in RT-qPCR analysis to investigate the tissue distribution of CTRP7 mRNA expression in mice. As shown in Figure 1(a), CTRP7 is widely expressed in various tissues *in vivo*, with the highest expression in fat, heart, and skeletal muscle, followed by kidney and liver, and a small amount in the hypothalamus.

To explore the impacts of IR and obesity on CTRP7 expression, we examined CTRP7 protein expression in the liver, fat, and skeletal muscle of ND- or HFD-fed WT and diabetic mice. CTRP7 protein expression in liver and adipose tissues was strongly elevated in HFD-fed, db/db, and ob/ob mice relative to ND-fed WT mice (Figures 1(b)–1(d)). In addition, CTRP7 expression in the skeletal muscle tissues was strongly decreased in HFD-fed and diabetic animals (Figures 1(b)–1(d)). These data indicate that CTRP7 may be related to obesity and diabetes.

### 3.2. Circulating CTRP7 and Adiponectin Levels in IGT and Newly Diagnosed T2DM Patients

The anthropometric and metabolic parameters in the study population were shown in Table 1. The results showed that in IGT individuals, obesity and metabolism-related indicators such as WHR, blood pressure (BP), triglyceride (TG), total cholesterol (TC), FFAs, HbA1c, FBG, 2-hour postglucose load blood glucose (2 h-BG), fasting insulin (FIns), 2-hour postglucose load blood insulin (2-Ins), area under the curve of glucose during oral glucose tolerance test (AUCg), and HOMA-IR were significantly increased, while serum ADIPOQ levels and HOMA-*β* were significantly decreased compared with NGT individuals (Table 1). In T2DM individuals, obesity-related parameters, BP, and biochemical indicators were further increased compared with IGT individuals, while high-density lipoprotein cholesterol (HDL-C), HOMA-*β*, and serum ADIPOQ levels were further decreased (Table 1).

**Figure 2:**
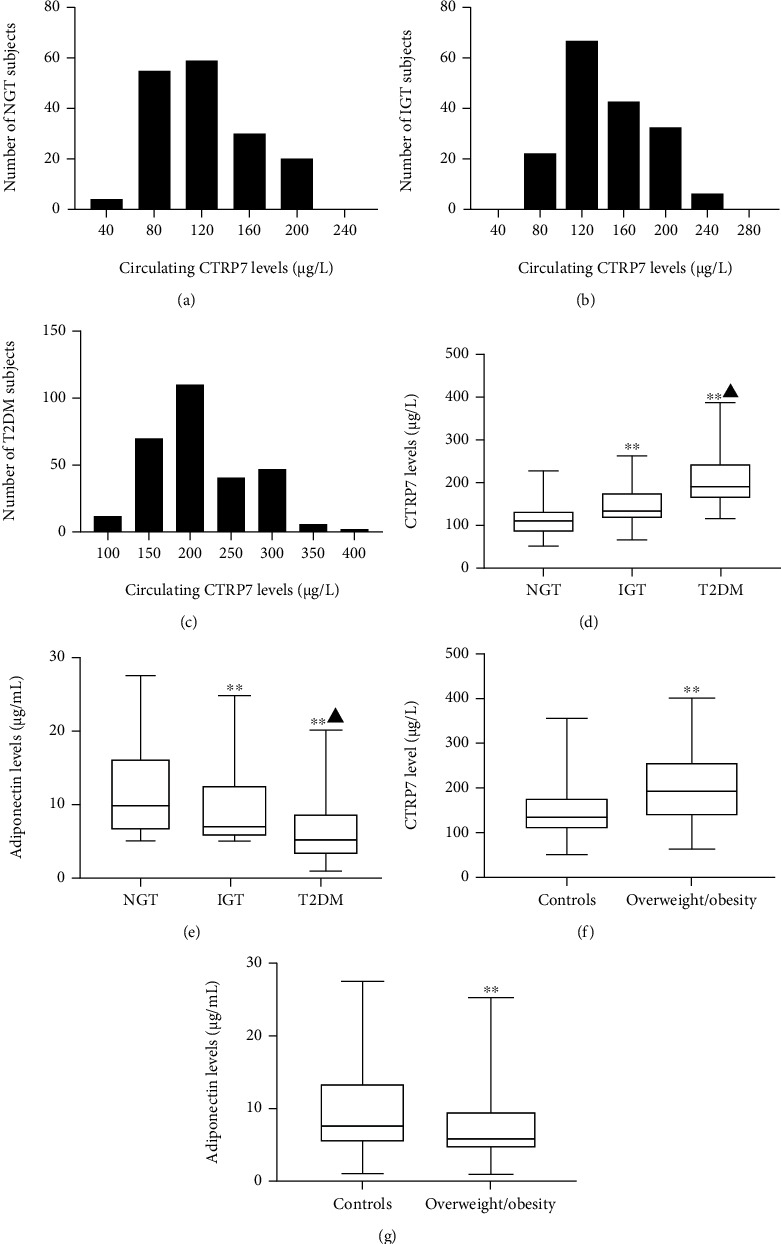
Circulating CTRP7 levels in the study population. (a)–(c) Distribution of circulating CTRP7 concentration in NGT (a), IGT (b), and T2DM (c) individuals. (d) Circulating CTRP7 levels in NGT, IGT, and T2DM individuals. (e) Circulating adiponectin levels in NGT, IGT, and T2DM individuals. (f) Circulating CTRP7 levels according to BMI (controls: BMI < 25 kg/m^2^; overweight/obese: BMI ≥ 25 kg/m^2^). (g) Circulating adiponectin levels according to BMI. NGT: normal glucose tolerance; IGT: impaired glucose tolerance; T2DM: type 2 diabetes mellitus. Box plot presentation, with the bottom and top of the box presenting the 25th and 75th percentiles, respectively, and the middle line presenting the median. ∗∗*p* < 0.01 vs. NGT or controls. ^▲^*p* < 0.05 vs. IGT.

As shown in Figure 2(a), the distribution of circulating CTRP7 concentration ranged from 113.2 to 125.6 *μ*g/L for the 95% NGT population. The distribution of serum CTRP7 concentration in IGT and T2DM individuals ranged from 137.7 to 149.5 *μ*g/L for the 95% IGT individuals and 203.2 to 216.4 *μ*g/L for 95% T2DM individuals, respectively (Figures 2(b) and 2(c)). Importantly, from IGT to T2DM patients, serum CTRP7 levels were significantly increased, while serum ADIPOQ levels (an insulin sensitizer) were visibly decreased compared with healthy individuals (Table 1 and Figures 2(d) and 2(e)).

We further divided all population into two groups (overweight/obese (W/O) group BMI ≥ 25 kg/m^2^ (*n* = 372) and normal group BMI < 25 kg/m^2^ (*n* = 252)) according to BMI. The levels of circulating CTRP7 in W/O individuals were notably higher than those in normal individuals (196.4 (148.9-256.4) vs. 147.2 (108.6-178.1) *μ*g/L, *p* < 0.01), while serum ADIPOQ levels were significantly lower (5.94 (4.61-9.88) vs. 7.56 (5.35-13.5) mg/L, *p* < 0.01) (Figures 2(f) and 2(g)). However, CTRP7 levels were comparable between men and women (data not shown).

### 3.3. Correlations between Circulating CTRP7 and Other Parameters as well as the Occurrence of IGT and T2DM

**Figure 3:**
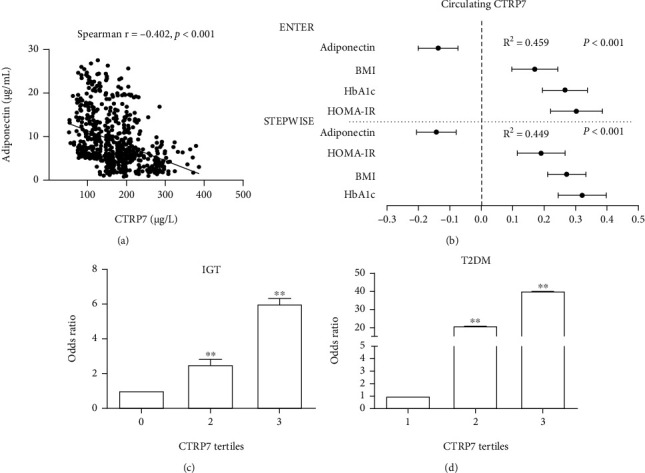
Association of circulating CTRP level with other variables, IGT and T2DM. (a) Correlations between circulating CTRP7 and adiponectin. (b) All factors and stepwise multiple regression analyses of the circulating CTRP7 in the study population. The circles correspond to the regression coefficients (ß), and the error bars indicate the 95% confidence interval (CI) of ß. (c) Prevalence of elevated IGT in different tertiles of CTRP7. (d) Prevalence of elevated T2DM in different tertiles of CTRP7. *R*^2^, coefficient of determination. Data are presented as mean ± SEM; ∗∗*p* < 0.01 vs. tertile 1.

We then investigate the relationship between serum concentrations of CTRP7 and ADIPOQ and other variables in all trial participants. Spearman correlation analysis found that serum CTRP7 was positively correlated with BMI, WHR, FAT %, BP, FBG, 2 h-BG, HbA1c, TG, TC, LDL-C, FFAs, AUCg, and HOMA-IR, while negatively correlated with HDL-C, HOMA-*β*, and ADIPOQ (Supplementary Table 2 and Supplementary Figure 3A). Multivariate correlation analyses revealed that BMI, HbA1c, HOMA-IR, and ADIPOQ were independently influential factors for serum CTRP7 (Supplementary Table 2). The equation of CTRP7 was Y_CTRP7_ = −92.09 + 0.274 × BMI + 0.321 × HbA1c + 0.191 × HOMA − IR–0.143 × ADIPOQ (*R* = 0.670, *R*^2^ = 0.449). Moreover, multivariate logistic regression found that even if some variables were controlled, CTRP7 is closely related to IGT and T2DM (Supplementary Table 3). When all participants were taken as a whole-body, including full factor and stepwise regression analysis, the main predictors of serum CTRP7 were ADIPOQ, HOMA-IR, BMI, and HbA1c (Figure 3(b)).

To further uncover the association of CTRP7 with IGT and T2DM, we divided CTRP7 concentrations into three tertiles in study population (tertile 1, <132.5 *μ*g/L; tertile 2, 132.5 to 195.2 *μ*g/L; and tertile 3, >195.2 *μ*g/L for T2DM; tertile 1, <108.3 *μ*g/L; tertile 2, 108.3 to 134.7 *μ*g/L; and tertile 3, >134.7 *μ*g/L for IGT). When serum CTRP7 levels were in tertile 2 and 3, the odds ratios (OR) of having IGT and T2DM were 2.55 (95% confidence interval (CI), 1.46; 4.43) and 6.06 (95% CI, 3.41; 10.8) for IGT, and 23.0 (95% CI, 13.2; 40.1) and 40.34 (95% CI, 21.6; 75.3) for T2DM (vs. tertile 1, *p* < 0.01; Figures 3(c) and 3(d)). Finally, to predict the occurrence of IGT and T2DM, we performed the ROC curve analysis. The area under the ROC curve for IGT (AUC_IGT_) was 0.69 with 54% specificity and 85% sensitivity (Supplementary Figure 1A). AUC_T2DM_ was 0.91 with 84% specificity and 87% sensitivity (Supplementary Figure 1B). The cut-off points for serum CTRP7 to predict IGT and T2DM were 105.2 *μ*g/L and 149.8 *μ*g/L, respectively.

### 3.4. Effects of OGTT, EHC, and Lipid-Induced IR on Serum CTRP7 in Healthy Humans

**Figure 4:**
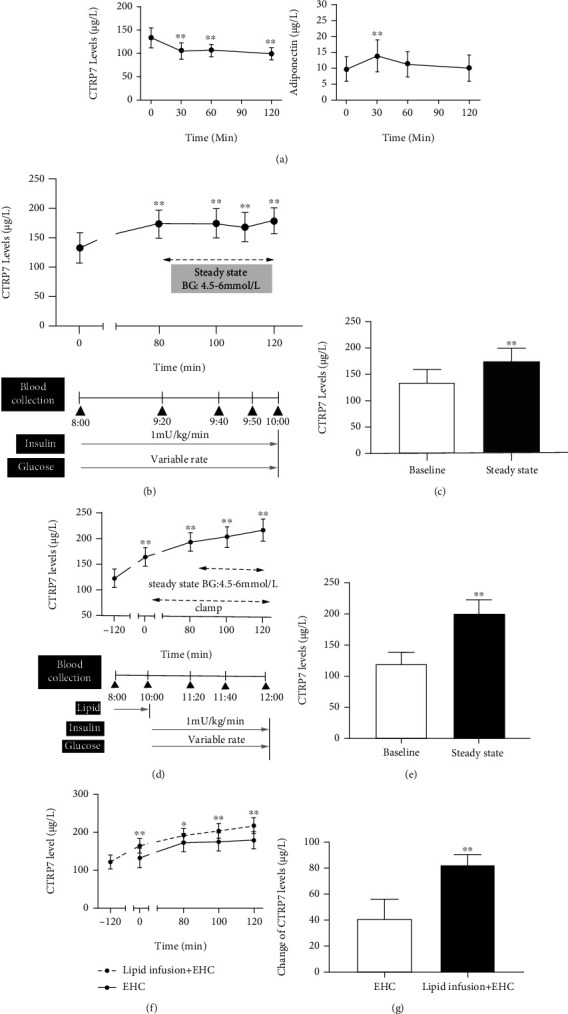
Circulating CTRP7 levels in interventional studies. (a) Circulating CTRP7 and adiponectin levels in healthy individuals during the OGTT. (b) Time course of circulating CTRP7 changes in healthy individuals during the EHC. (c) CTRP7 levels at baseline and the steady-state of the EHC. (d) Time course of circulating CTRP7 changes in healthy individuals during lipid infusion combined with EHC. (e) Circulating CTRP7 levels at baseline and the steady-state of lipid infusion combined with EHC. (f) Time course of serum CTRP7 changes during the EHC alone and lipid perfusion combined with EHC. (g) Changes of circulating CTRP7 levels at the steady-state of the EHC alone and lipid infusion combined with EHC. BG: blood glucose. Data are presented as mean ± SD; ∗*p* < 0.05 or ∗∗*p* < 0.01 vs. baseline or EHC.

Subsequently, we examined the effect of nutritional status on circulating CTRP7 in healthy individuals by an OGTT trial. During the OGTT, circulating ADIPOQ levels showed a transient increase due to elevated blood glucose and insulin caused by oral glucose challenge (from 9.78 ± 3.91 to 13.9 ± 5.07 mg/L at 30 min, then to 11.3 ± 4.04 mg/L at 60 min, and 10.1 ± 4.11 mg/L at 120 min, Figure 4(a)). However, during the OGTT, circulating CTRP7 concentration gradually decreased from 134.6 ± 21.2 to 105.9 ± 17.6 *μ*g/L at 30 min, then to 105.5 ± 14.1 *μ*g/L at 60 min, and finally to 99.9 ± 13.9 *μ*g/L at 120 min (Figure 4(a)), indicating that hyperglycemia and/or hyperinsulinemia inhibited the secretion and release of CTRP7.

To further investigate the impact of elevated insulin levels on the secretion of CTRP7, we performed an EHC experiment in healthy individuals. During the EHC, blood glucose was clamped at 4-6 mmol/L, while insulin level was gradually increased (from 8.0 ± 3.0 to 105.6 ± 23.1 mU/L). During the EHC experiment, the serum levels of CTRP7 increased significantly with the increase of insulin levels from 132.9 ± 25.8 to 173.2 ± 24.0 *μ*g/L at 80 min, then to 175.3 ± 23.9 *μ*g/L at 100 min, and finally to 179.3 ± 22.8 *μ*g/L at 120 min (Figures 4(b) and 4(c)), indicating that hyperinsulinemia promotes CTRP7 secretion *in vivo*.

To further explore the relationship between CTRP7 and IR, we conducted a lipid-infusion test combined with the EHC in healthy individuals. During the stable period of EHC, lipid infusion resulted in a significant decrease in the GIR and *M*-values, indicating an acute-IR *in vivo* (Supplementary Figure 2A and 2B).

As shown in Figures 4(d) and 4(e), lipid-infusion markedly increased serum CTRP7 concentrations compared with the baseline levels (from 122.3 ± 18.3 to 204.7 ± 21.9 *μ*g/L, Figures 4(d) and 4(e)). In addition, compared with EHC alone, lipid-infusion combined with EHC further increased circulating CTRP7 levels (82.4 ± 8.25 vs. 41.1 ± 15.2 *μ*g/L, *p* < 0.01; Figures 4(f) and 4(g)). These data indicate that serum CTRP7 levels are upregulated by serum FFAs and insulin levels.

### 3.5. Effects of Physical Activity and Cold Stimulation on Circulating CTRP7 in Humans and Mice

**Figure 5:**
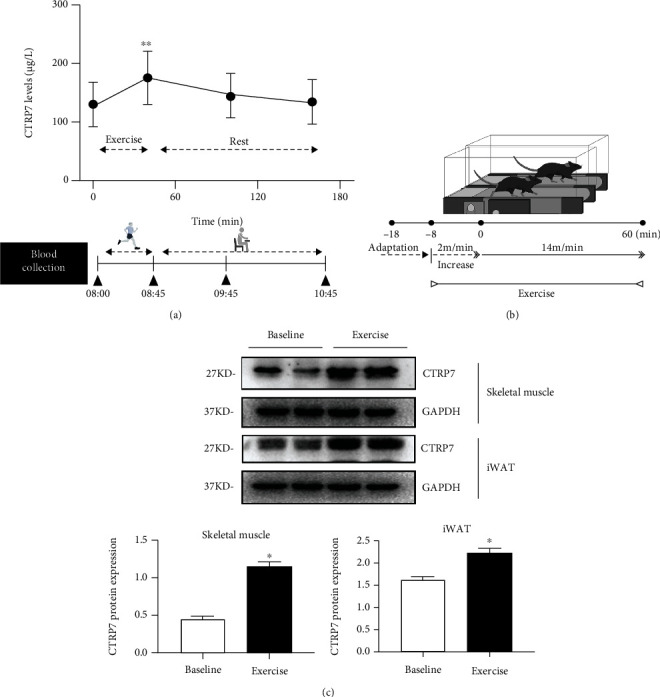
Effects of physical activity on the circulating level and protein expression of CTRP7 in humans and mice. (a) Circulating CTRP7 levels in response to a 45 min bout of physical activity in healthy individuals. (b) The schematic diagram of experimental design for exercise test in mice. (c) CTRP7 protein expression of iWAT and skeletal muscle in response to a 60 min treadmill exercise in mice (*n* = 3). iWAT: inguinal adipose tissue. Data are presented as mean ± SD or SEM; ∗*p* < 0.05 or ∗∗*p* < 0.01 vs. baseline.

To elucidate the effects of energy metabolism on the secretion and release of CTRP7 *in vivo,* we performed physical activity and cold-stimulation experiments in humans. Acute physical exercise for 45 min resulted in a significant increase in circulating CTRP7 concentration (from 129.1 ± 39.0 to 174.9 ± 45.1 *μ*g/L; *p* < 0.01, Figure 5(a)). After the exercise, CTRP7 concentration gradually decreased (from 174.9 ± 45.1 to 144.9 ± 38.0 *μ*g/L), finally returned to baseline level at 120 min after rest (Figure 5(a)). Next, we investigated the effect of body temperature changes on the release of CTRP7 in humans. We found that there was no significant change in serum CTRP7 concentration in healthy humans before and after cold exposure, indicating that cold stress did not stimulate the release of CTRP7 from adipose tissues (Supplementary Figure 3A and 3B). These results may indicate that physical activity, but not thermogenesis caused by cold, leads to increased CTRP7 secretion.

To further study the effect of physical activity on CTRP7 in animals, we performed a 60 min treadmill experiment in mice and measured the expression of CTRP7 protein in both skeletal muscle and adipose tissues (Figure 5(b)). As shown in Figure 5(c), CTRP7 protein expression in the iWAT and skeletal muscles of mice was significantly elevated after acute treadmill exercise. This finding further indicates that physical activity increases the synthesis of CTRP7 protein in adipose and skeletal muscle tissues.

### 3.6. Effects of Glucose, Insulin, and FFAs on CTRP7 Expression In Vitro

**Figure 6:**
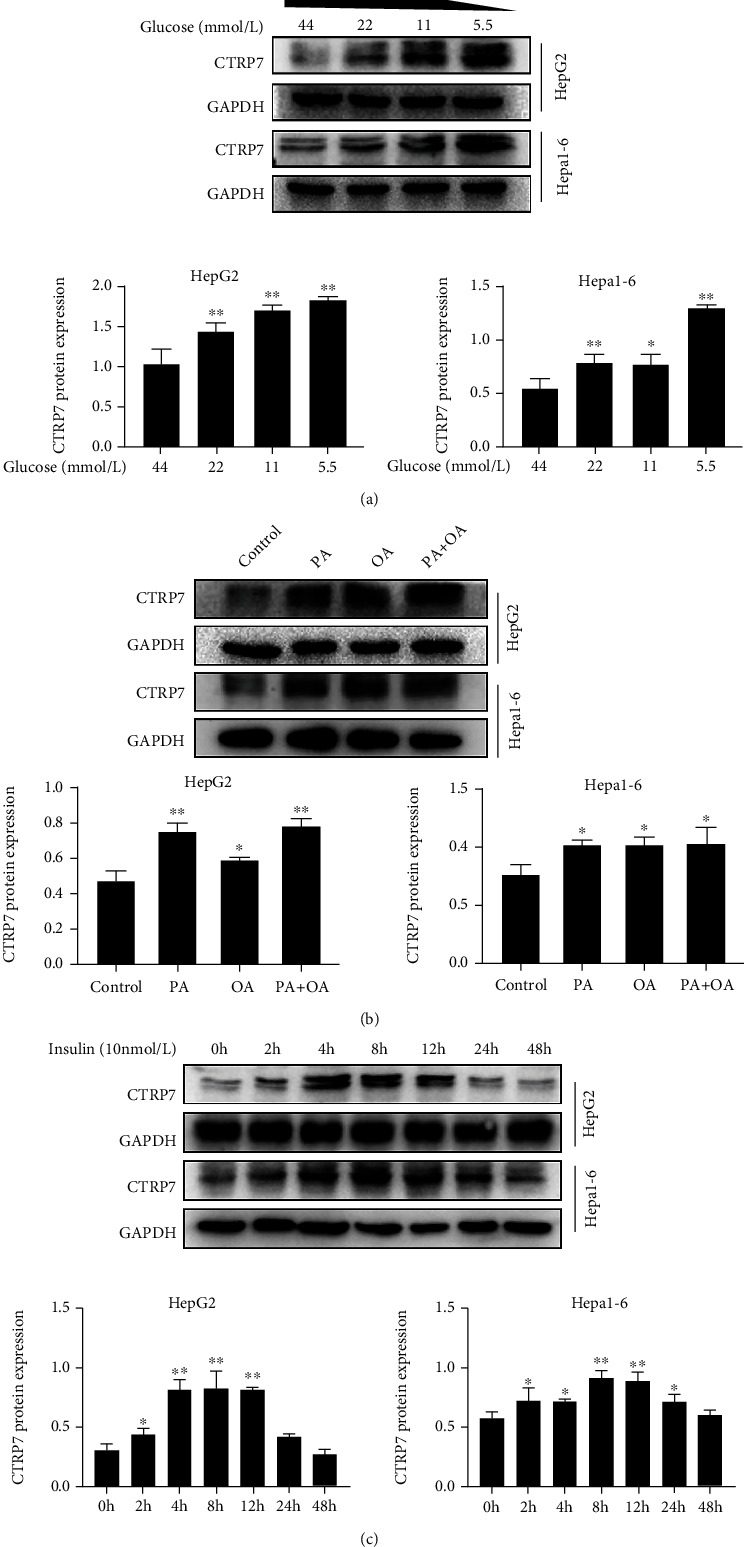
Effects of glucose, fatty acid, and insulin on the expression of CTRP7 in hepatocytes. (a) CTRP7 protein expression in HepG2 and Hepa1-6 cells treated with different concentrations of glucose for 24 h. (b) CTRP7 protein expression in HepG2 and Hepa1-6 cells treated with palmitic, oleate acid, FFAs, or BSA for 24 h. (c) CTRP7 protein expression in HepG2 and Hepa1-6 cells treated with insulin (10 nM) for 2-48 h. Data are presented as mean ± SEM; ∗*p* < 0.05 or ∗∗*p* < 0.01 vs. high glucose group, controls or 0 h.

To further explore the effects of nutritional status and hormone on CTRP7 expression at the cellular levels, we treated HepG2 and Hepa1-6 cells with different concentrations of glucose, palmitic acid, oleic acid, FFAs, or insulin, and then measured the levels of CTRP7 protein. We found that the expression of CTRP7 protein in both HepG2 and Hepa1-6 cells was gradually increased with the decrease of glucose concentration (Figure 6(a)). Furthermore, treatment with fatty acid significantly increased the expression of CTRP7 protein in these cells (Figure 6(b)). To investigate the effect of prolonged insulin stimulation on CTRP7 expression *in vitro,* hepatocytes were stimulated with insulin for 2 to 48 h. The results showed that insulin increased CTRP7 protein levels by ~15% to 60% that persisted for 2 to 12 hours in HepG2 cells. In Hepa1-6 cells, the expression of CTRP7 protein also increased to a certain extent (Figure 6(c)). These *in vitro* results are consistent with intervention studies *in vivo*, indicating that the production of CTRP7 is regulated by glucose, fatty acids, and insulin.

### 3.7. CTRP7 Overexpression Facilitated Gluconeogenic Gene Expression and Oxidative Stress and Inhibited Insulin Signaling in Hepa1-6 Cells

**Figure 7:**
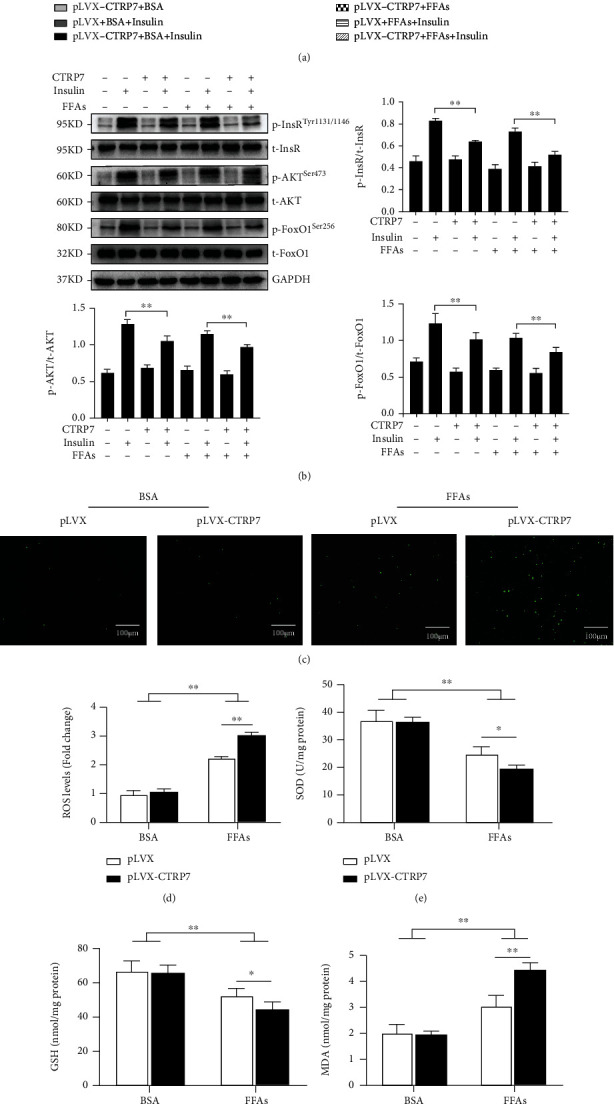
CTRP7 overexpression facilitates insulin resistance and oxidative stress. Hepa1-6 cells were transfected with pLVX-Puro or pLVX-*CTRP7* for 24 h and then exposed BSA or FFAs for another 24 has indicated in the methods. (a) mRNA expression of genes related to glucose metabolism. (b) Effect of CTRP7 overexpression on phosphorylation of insulin signaling molecules. (c) Intracellular ROS is shown with DCFH staining (green). (d) Quantitative analysis of DCFH-DA staining. (e) Intracellular SOD activity. (f) Intracellular GSH levels. (g) Intracellular MDA content. Data are presented as mean ± SEM; ∗*p* < 0.05 or ∗∗*p* < 0.01.

To further explore CTRP7's influence on metabolism and insulin signal pathway, Hepa1-6 cells were infected with pLVX-*CTRP7*, a plasmid expressing CTRP7, or control plasmid (pLVX-Puro), then treated with FFAs and insulin. Genes related to glucose metabolism and insulin signals were analyzed by western blotting. As expected, CTRP7 expression at mRNA and protein levels was notably increased in pLVX-*CTRP7*-transfected Hepa1-6 cells (Supplementary Figure 4). We found that in pLVX-*CTRP7*-transfected cells, the mRNA expression of gluconeogenesis and glycogen breakdown related genes, including PEPCK, G6Pase, and Pygl, increased significantly (Figure 7(a)), while the expression of glycolysis and glycogen synthesis related genes (Gck and Gys2) decreased significantly (Figure 7(a)). In addition, overexpression of CTRP7 resulted in a significant reduction in the phosphorylation levels of insulin signaling molecules, including phospho-InsR^Tyr1150^, phospho-Akt^ser473^, and phosphor-FoxO1^ser256^ (Figure 7(b)). These results are consistent with *in vivo* studies, which further indicate that CTRP7 promotes glucose-metabolism disorder and IR.

It has been demonstrated that metabolic disorder and IR is associated with oxidative stress [21]. Therefore, we next observed the influence of CTRP7 overexpression on FFA-caused oxidative stress in Hepa1-6 cells. DCFH-DA staining showed that overexpression of CTRP7 resulted in significant enhancement of intracellular ROS signal (green) in FFA-treated Hepa1-6 cells (Figure 7(c)). Furthermore, the determination of antioxidant enzyme activity showed that the activities of SOD and GSH decreased significantly, while ROS activity and MDA content significantly increased (Figures 7(d)–7(g)). These data pointed out that CTRP7 reinforced oxidative stress in FFA-treated Hepa1-6.

## 4. Discussion

In recent years, some CTRP family proteins, such as CTRP5, CTRP6, and CTRP15, have been found to be associated with metabolic disorders, but there are very few reports about CTRP7 and its association with metabolic diseases [22–24]. In particular, the relationship of CTRP7 with IR and T2DM remains unknown. In the present study, we have examined circulating CTRP7 levels in IGT and T2DM patients and healthy controls. Our results revealed that, contrary to the findings for ADIPOQ (an insulin sensitizer), the levels of circulating CTRP7 were markedly increased in individuals with IGT and T2DM. The data indicate that the secretion and release of CTRP7 gradually increase with the progression of metabolic disorder from IGT to T2DM. Additionally, correlation analysis showed that CTRP7 is positively correlated with obesity, glucose level, lipid level, and IR and is an independent influential factor for IGT and T2DM. In individuals with IGT and T2DM, circulating CTRP7 levels showed an opposite trend to circulating ADIPOQ levels; thus, the increase in CTRP7 levels may represent a compensatory response to decreased ADIPOQ levels or the stimulation of IR and metabolic disorder. Our results are similar to those of a previous study (*n* = 37) which showed that circulating CTRP7 levels were elevated in individuals with obesity [11]. Thus, both studies demonstrate a physiological link between CTRP7 and obesity, as well as IR, in humans. Furthermore, in the animal studies conducted in the present study, the level of CTRP7 protein in the liver and fat of HFD-fed mice and diabetic mice showed a pronounced increase, while it was significantly decreased in skeletal muscle tissues. Similarly, a study in ob/ob mice also reported upregulated CTRP7 mRNA expression in adipose tissues [9]. Another study showed that in HFD-fed mice, the expression of CTRP7 mRNA in the liver was significantly upregulated, while it was downregulated in muscle tissue and unchanged in adipose tissues [11]. The reason for the tissue-dependent difference in CTRP7 expression is unclear, but it indicates that CTRP7 overproduction under obesity and IR states may take place mainly in the liver and fatty tissue. Altogether, these data indicate that under the pathological conditions of IR, the local expression and impact of CTRP7 may vary with the metabolic environment. Thus, CTRP7 may play a role in mediating cross-talk between cells and tissues, and importantly, CTRP7 protein expression might be tissue-specific.

Consistent with our *in vivo* findings, our *in vitro* findings showed that CTRP7 overexpression facilitated the mRNA expression of gluconeogenic genes and suppressed the phosphorylation of InsR, Akt, and FoxO1. Accordingly, Petersen et al. found that in CTRP7-knockout mice, CTRP7 deficiency improved glucose tolerance and IR under HFD feeding conditions [11]. Therefore, our *in vivo* and *in vitro* studies further confirm that CTRP7 promotes metabolism disorder and IR.

To elucidate the effects of nutritional status and insulin levels on the secretion and release of CTRP7, we observed the dynamic changes in serum CTRP7 level in response to a glucose challenge and an EHC in control participants. The healthy participants were included to eliminate the potential effect of comorbidities or metabolic derangements. We found that in response to the OGTT, serum CTRP7 concentration gradually decreased. This is probably because both the glucose and insulin levels significantly increased during OGTT. Based on these findings, it is not clear whether glucose or insulin or both glucose and insulin inhibit the secretion of CTRP7 *in vivo*. To address this issue, we performed an EHC test, which is the gold standard for evaluating IR and glucose metabolism, to clamp glucose at the base level and observe changes in serum CTRP7 under high-insulin conditions. We found that hyperinsulinemia caused a significant increase in circulating CTRP7 levels when blood glucose was clamped at basal levels; thus, hyperinsulinemia may promote the secretion of CTRP7 *in vivo*. Further, in our *in vitro* experiments, we found that CTRP7 protein expression exhibited a glucose concentration-dependent reduction and an insulin-induced increase that persisted for 2 to 48 h in hepatocytes. Therefore, based on the results of OGTT and EHC as well as the *in vitro* studies, we can conclude that hyperglycemia inhibits, while hyperinsulinemia stimulates, the secretion of CTRP7 *in vivo.*

Many studies have shown that increasing levels of FFAs caused by lipid infusion can affect insulin sensitivity and induce acute IR *in vivo* [25–27]. Therefore, to further examine the relationship between CTRP7 and IR, we conducted an experiment in which lipid infusion combined with EHC was used to build an acute IR model for evaluating the effect of change in nutritional status on circulating CTRP7 levels in normal populations. Our results show that serum CTRP7 levels were significantly increased in normal individuals during lipid infusion in response to acute IR caused by elevated FFA levels. Similarly, in our *in vitro* study, we observed that FFA treatment led to a significant increase in the expression of CTRP7 protein in hepatocytes. Taken together, these intervention studies and *in vitro* studies show that FFAs and insulin are the two main regulators of elevated CTRP7 levels. Given that FFA and insulin levels are elevated in T2DM and IGT individuals, both FFA- and insulin-induced effects might be relevant to the pathogenesis of increased CTRP7 levels under *in vivo* conditions.

It has been well established that physical activity can improve glucose and lipid metabolism and ameliorate IR [28, 29], as well as regulate the secretion of myokines. So far, only a few myokines, such as IL-6, IL-15, and BDNF, have been confirmed to be regulated by exercise [15, 30, 31]. To investigate whether acute exercise can regulate the secretion and release of CTRP7, we performed an acute exercise intervention in healthy individuals and observed that the circulating concentrations of CTRP7 were raised. Similarly, a 60 min treadmill experiment led to a significant increase in CTRP7 protein expression in the skeletal muscle and iWAT in mice. These data show that CTRP7 may also be a myokine. However, the reason for the increase in CTRP7 expression and circulating levels during physical activity is unclear. Previous studies have shown that acute exercise can increase serum FFA and cortisol levels [32]. Therefore, the exercise-induced increase in CTRP7 expression could be associated with an increase in FFAs and cortisol.

Cold exposure induces thermogenesis and increases both brown adipose tissue quantity and activity *in vivo* [33, 34]; this leads to not only weight loss but also improvements in lipid and glucose homeostasis, as well as insulin sensitivity [35, 36]. Herein, we studied the effect of cold stimulation on serum CTRP7 levels in adult individuals. We observed that short-term cold stimulation did not change the *in vivo* concentrations of serum CTRP7. This indicates that the activation of brown adipose tissue in response to acute exposure to cold environments does not affect the production of CTRP7. Therefore, CTRP7 may not play a role in the process of thermogenesis under *in vivo* conditions. However, as the cold exposure was only for a short duration in our experiment, the finding needs to be confirmed under conditions of longer cold exposure.

It has been demonstrated that oxidative stress caused by metabolic disorder is one of the important causes of IR, and it is also considered to be an important mechanism of IR [37]. It has also been reported that CTRP7 deficiency suppressed oxidative and endoplasmic reticulum stress in obese animals [11]. In the current study, we found that overexpression of CTRP7 facilitated FFA-induced oxidative stress in hepatocytes. Therefore, we believe that CTRP7 leads to IR by, at least in part, promoting oxidative stress induced by metabolic disorder. However, further research is needed to address the mechanism by which CTRP7 promotes oxidative stress.

Some limitations of the present research must be acknowledged: (1) this is a cross-sectional population-based, intervention study that was conducted under *in vivo* and *in vitro* settings. Therefore, a causal relationship between CTRP7 and T2DM cannot be inferred. (2) As this study was conducted on a Chinese population sample, the results should not be generalized to other populations. (3) Despite the relatively large sample size, the number of IGT and T2DM patients was still very limited. Therefore, it is necessary to have larger study cohorts to confirm these findings. In addition, the mechanism of CTRP7 transcriptional regulation by nutrients and hormones remains to be studied. Nonetheless, we still believe that as a pilot study, it has important guiding significance for further research on CTRP7 and metabolic diseases.

In conclusion, this study demonstrates the changes in circulating CTRP7 levels and their relationship with IR and energy metabolism in populations with different levels of glucose tolerance. In addition, the possible regulatory factors of circulating CTRP7, including glucose, insulin, and FFAs, were verified by various intervention and animal studies as well as *in vitro* experiments. Overall, these findings imply that CTRP7 may be a new metabolic marker and drug target and lay an important foundation for future research on the role of CTRP7 in metabolic diseases.

## Figures and Tables

**Table 1:** Main clinical features in the study population.

Variable	NGT	IGT	nT2DM
Age (yr)	54.0 ± 6.0	54.3 ± 10.8	55.2 ± 9.9
Sex (M/F)	168 (82/86)	168 (81/87)	288 (147/141)
BMI (kg/m^2^)	23.5 ± 2.21	24.8 ± 2.50∗∗	25.4 ± 2.87∗∗^▲^
FAT (%)	27.3 ± 4.24	28.4 ± 3.90∗	30.1 ± 6.26∗∗^▲▲^
WHR	0.86 ± 0.05	0.89 ± 0.04∗∗	0.90 ± 0.05∗∗
SBP (mmHg)	124.1 ± 17.2	132.8 ± 18.2∗∗	133.0 ± 16.8∗∗^▲^
DBP (mmHg)	79.2 ± 10.6	81.5 ± 10.7	83.0 ± 11.5∗∗^▲▲^
TC (mmol/L)	4.75 ± 0.75	4.92 ± 0.89∗	5.15 ± 0.50∗∗^▲▲^
TG (mmol/L)	1.32 (0.91-1.79)	1.90 (1.47-2.47)∗∗	2.01 (1.73-2.33)∗∗^▲^
HDL-C (mmol/L)	1.38 ± 0.34	1.40 ± 0.29	1.21 ± 0.12∗∗^▲▲^
LDL-C (mmol/L)	2.54 ± 0.65	2.60 ± 0.59	3.00 ± 0.22∗∗^▲▲^
FFAs (*μ*mol/L)	0.43 (0.36-0.57)	0.54 (0.46-0.69)∗∗	0.66 (0.59-0.73)∗∗^▲▲^
HbA1c (%)	5.48 ± 0.44	5.79 ± 0.46∗∗	8.30 ± 1.05∗∗^▲▲^
FBG (mmol/L)	5.04 ± 0.33	5.89 ± 0.49∗∗	7.17 ± 0.77∗∗^▲▲^
2 h-BG (mmol/L)	6.29 ± 1.04	8.31 ± 1.04∗∗	12.8 ± 1.28∗∗^▲▲^
FIns (mU/L)	8.59 (7.85-9.18)	9.23 (8.18-10.5)∗∗	12.1 (9.60-14.3)∗∗^▲▲^
2 h-Ins (mU/L)	40.9 (37.7-46.9)	58.5 (50.8-65.2)∗∗	53.8 (45.7-63.9)∗∗^▲▲^
AUCg	14.7 ± 2.13	19.1 ± 1.77∗∗	30.1 ± 4.39∗∗^▲▲^
AUCi	92.9 ± 6.59	128.4 ± 10.8∗∗	84.0 ± 11.4∗∗^▲▲^
HOMA-IR	1.93 (1.73-2.11)	2.37 (2.08-2.81)∗∗	3.77 (2.94-4.56)∗∗^▲▲^
HOMA-*β*	109.7 (93.2-129.5)	77.4 (67.3-90.3)∗∗	67.4 (52.0-79.7)∗∗^▲▲^
CTRP7 (*μ*g/L)	111.8 (86.7-147.7)	133.0 (112.9-177.9)∗∗	196.4 (168.1-254.7)∗∗^▲▲^
Adiponectin (*μ*g/mL)	9.86 (6.49-16.2)	7.01 (5.62-12.6)∗∗	5.24 (3.18-8.74)∗∗^▲▲^

BMI: body mass index; FAT (%): the percentage of fat *in vivo*; WHR: waist-hip ratio; SBP: systolic blood pressure; DBP: diastolic blood pressure; TC: total cholesterol; TG: triglyceride; HDL-C: high-density lipoprotein cholesterol; LDL-C: low-density lipoprotein cholesterol; FFAs: free fatty acid; HbA1c: hemoglobin A1c; FBG: fasting blood glucose; 2 h-BG: 2 h postglucose load blood glucose; FIns: fasting plasma insulin; 2 h-Ins: 2 h postglucose load blood insulin; AUCg: area under curve of glucose during OGTT; AUCi: area under curve of insulin during OGTT; HOMA-IR: homeostasis model assessment of insulin resistance; HOMA-*β*: an index of *β*-cell functions; values are means ± SD or median (interquartile range). ∗*p* < 0.05, ∗∗*p* < 0.01 vs. NGT; ^▲^*p* < 0.05, ^▲▲^*p* < 0.01 vs. IGT.

## Data Availability

Data are available on request.

## Abbreviations

CTRP: C1q/tumor necrosis factor-related proteins
IR: Insulin resistance
OGTT: Oral glucose tolerance test
EHC: Euglycemic-hyperinsulinemic clamp
IGT: Impaired glucose tolerance
T2DM: Type 2 diabetes mellitus
HOMA-IR: Homeostasis model assessment of IR
FFAs: Free fatty acids
iWAT: Inguinal white adipose tissue
ROS: Reactive oxygen species
SOD: Superoxide dismutase
GSH: Glutathione
MDA: Malondialdehyde.

## Supplementary Materials

Supplementary Materials Include Table S1-S3, Figure S1-S4. Supplementary Table 1: specific primers used for RT-PCR analyses. Supplementary Table 2: the correlation analysis of serum CTRP7 levels with other variables in study population. Supplementary Table 3: association of circulating CTRP7 levels with IGT and nT2DM in fully adjusted models. Supplementary Figure 1: ROC curve analyses were performed for the prediction of IGT (a) and T2DM (b). Supplementary Figure 2: lipid infusion induced insulin resistance in vivo. Supplementary Figure 3: changes of serum CTRP7 level in a cold-exposure test. Supplementary Figure 4: CTRP7 expression at mRNA and protein levels in Hepa1-6 cells.
