# Supplementary figures and images for: CTRP7 Is a Biomarker Related to Insulin Resistance and Oxidative Stress: Cross-Sectional and Intervention Studies In Vivo and In Vitro
Source: Oxid Med Cell Longev. 2022 Mar 23;2022:6877609. doi: 10.1155/2022/6877609 (PMC8967592; doi:10.1155/2022/6877609)

A

## ROC curve

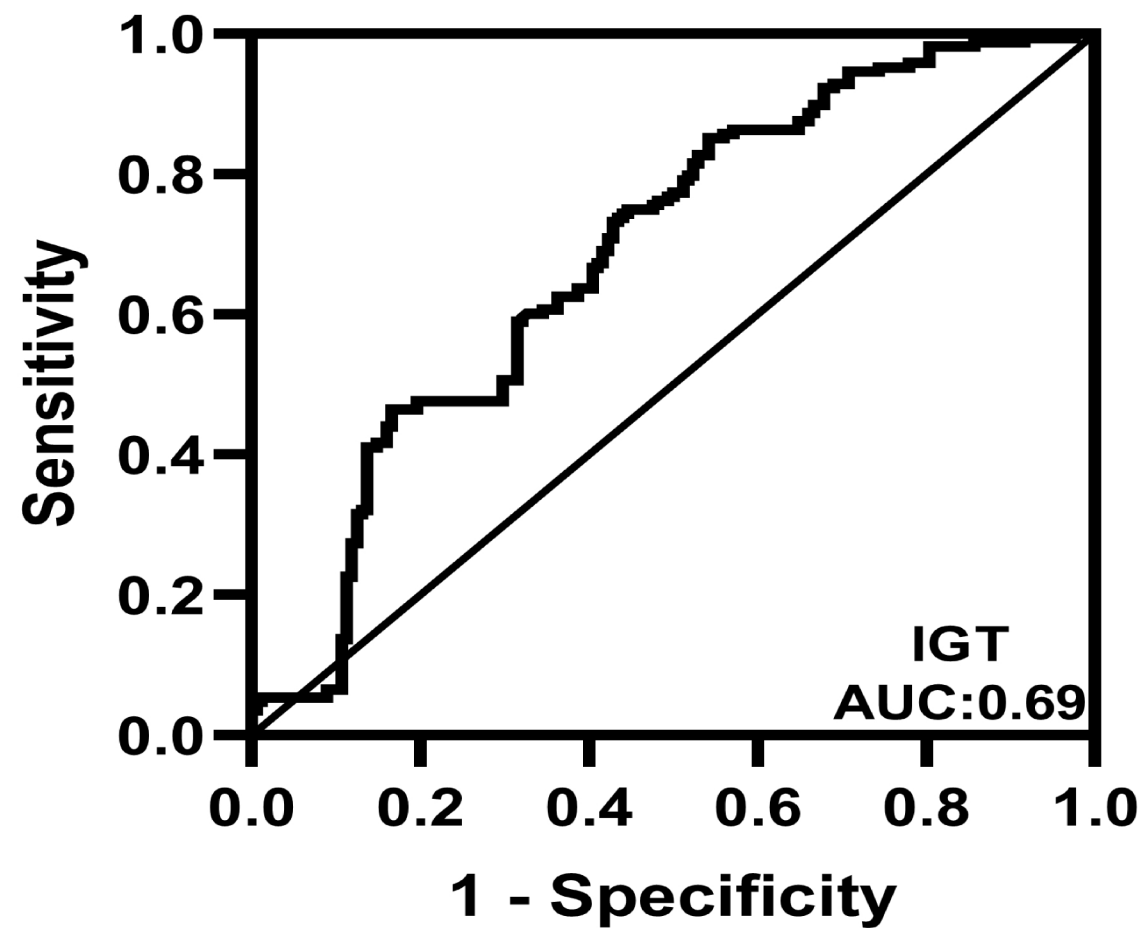

B

## ROC curve

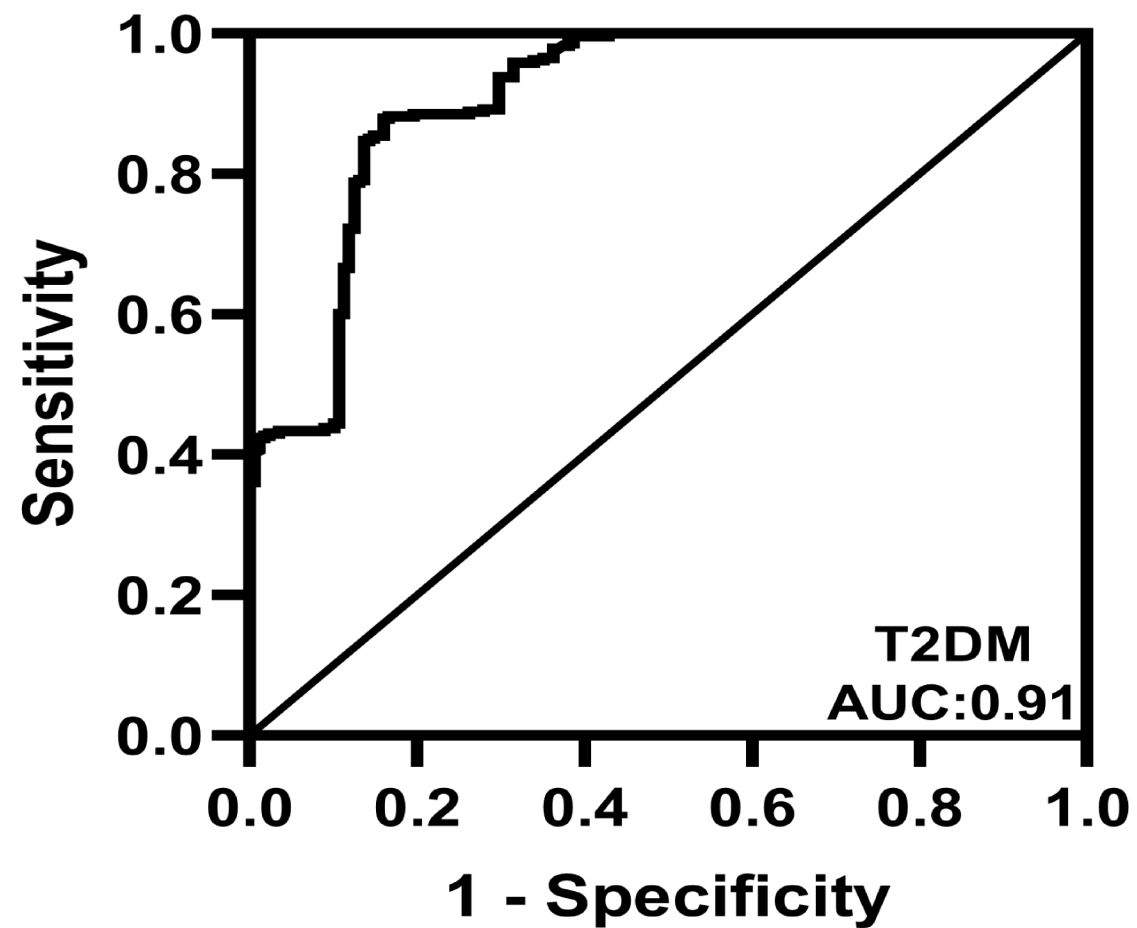

Supplement: Supplementary Materials — Include Table S1-S3, Figure S1-S4. Supplementary Table 1: specific primers used for RT-PCR analyses. Supplementary Table 2: the correlation analysis of serum CTRP7 levels with other variables in study population. Supplementary Table 3: association of circulating CTRP7 levels with IGT and nT2DM in fully adjusted models. Supplementary Figure 1: ROC curve analyses were performed for the prediction of IGT (a) and T2DM (b). Supplementary Figure 2: lipid infusion induced insulin resistance in vivo. Supplementary Figure 3: changes of serum CTRP7 level in a cold-exposure test. Supplementary Figure 4: CTRP7 expression at mRNA and protein levels in Hepa1-6 cells. [file 6877609.f1.zip › Supplementary Fig 1 (1).pdf]

**A**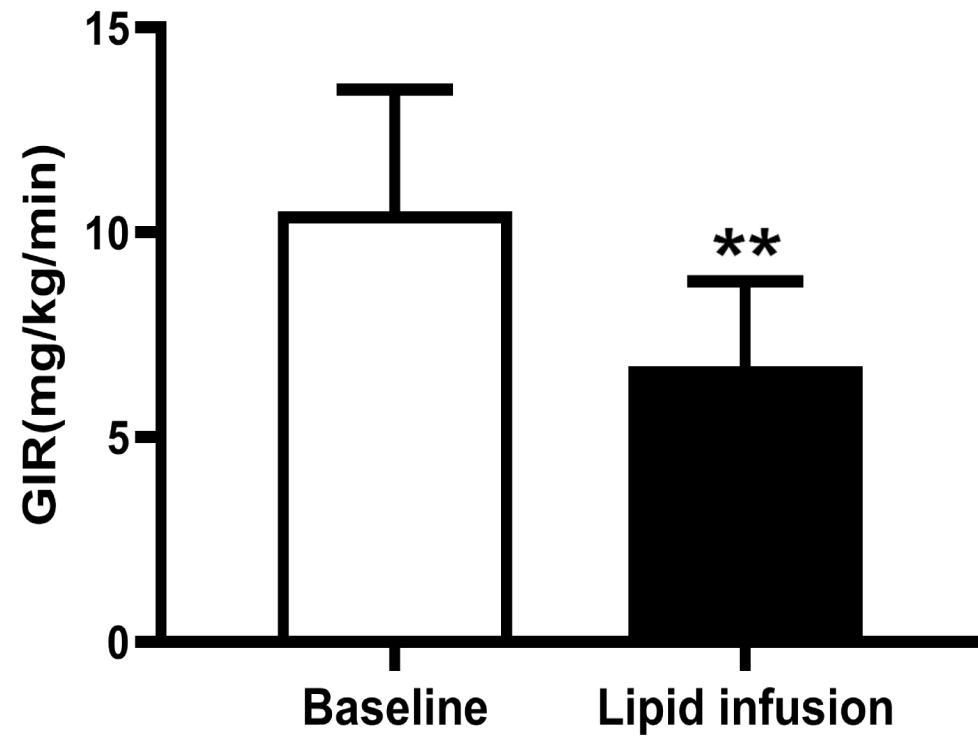**B**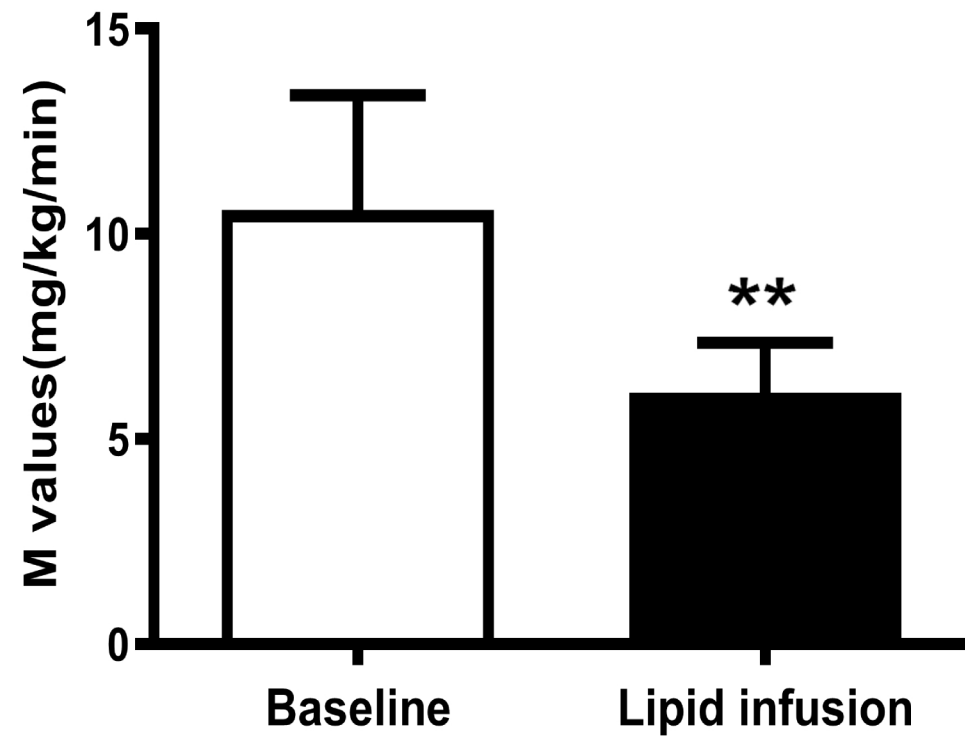

Supplement: Supplementary Materials — Include Table S1-S3, Figure S1-S4. Supplementary Table 1: specific primers used for RT-PCR analyses. Supplementary Table 2: the correlation analysis of serum CTRP7 levels with other variables in study population. Supplementary Table 3: association of circulating CTRP7 levels with IGT and nT2DM in fully adjusted models. Supplementary Figure 1: ROC curve analyses were performed for the prediction of IGT (a) and T2DM (b). Supplementary Figure 2: lipid infusion induced insulin resistance in vivo. Supplementary Figure 3: changes of serum CTRP7 level in a cold-exposure test. Supplementary Figure 4: CTRP7 expression at mRNA and protein levels in Hepa1-6 cells. [file 6877609.f1.zip › Supplementary Fig 2 (1).pdf]

A

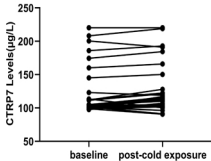

B

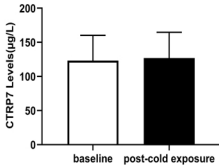

Supplement: Supplementary Materials — Include Table S1-S3, Figure S1-S4. Supplementary Table 1: specific primers used for RT-PCR analyses. Supplementary Table 2: the correlation analysis of serum CTRP7 levels with other variables in study population. Supplementary Table 3: association of circulating CTRP7 levels with IGT and nT2DM in fully adjusted models. Supplementary Figure 1: ROC curve analyses were performed for the prediction of IGT (a) and T2DM (b). Supplementary Figure 2: lipid infusion induced insulin resistance in vivo. Supplementary Figure 3: changes of serum CTRP7 level in a cold-exposure test. Supplementary Figure 4: CTRP7 expression at mRNA and protein levels in Hepa1-6 cells. [file 6877609.f1.zip › Supplementary Fig 3 (1).pdf]

**A**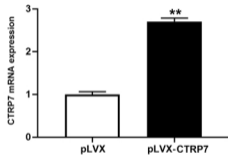**B**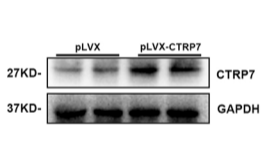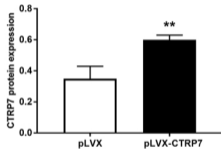

Supplement: Supplementary Materials — Include Table S1-S3, Figure S1-S4. Supplementary Table 1: specific primers used for RT-PCR analyses. Supplementary Table 2: the correlation analysis of serum CTRP7 levels with other variables in study population. Supplementary Table 3: association of circulating CTRP7 levels with IGT and nT2DM in fully adjusted models. Supplementary Figure 1: ROC curve analyses were performed for the prediction of IGT (a) and T2DM (b). Supplementary Figure 2: lipid infusion induced insulin resistance in vivo. Supplementary Figure 3: changes of serum CTRP7 level in a cold-exposure test. Supplementary Figure 4: CTRP7 expression at mRNA and protein levels in Hepa1-6 cells. [file 6877609.f1.zip › Supplementary Fig 4 (1).pdf]
