# Supplementary material for: CTRP7 Is a Biomarker Related to Insulin Resistance and Oxidative Stress: Cross-Sectional and Intervention Studies In Vivo and In Vitro
Source: Oxid Med Cell Longev. 2022 Mar 23;2022:6877609. doi: 10.1155/2022/6877609 (PMC8967592; doi:10.1155/2022/6877609)
Supplement: Supplementary Materials — Include Table S1-S3, Figure S1-S4. Supplementary Table 1: specific primers used for RT-PCR analyses. Supplementary Table 2: the correlation analysis of serum CTRP7 levels with other variables in study population. Supplementary Table 3: association of circulating CTRP7 levels with IGT and nT2DM in fully adjusted models. Supplementary Figure 1: ROC curve analyses were performed for the prediction of IGT (a) and T2DM (b). Supplementary Figure 2: lipid infusion induced insulin resistance in vivo. Supplementary Figure 3: changes of serum CTRP7 level in a cold-exposure test. Supplementary Figure 4: CTRP7 expression at mRNA and protein levels in Hepa1-6 cells. [file 6877609.f1.zip › Supplemental Material (1).doc]

**Supplementary Table 1.** Specific primers used for RT-PCR analyses

| Gene | Forward (5’-3’) | Reverse (5’-3’) |
| --- | --- | --- |
| Pepck | CTGCATAACGGTCTGGACTTC | GCCTTCCACGAACTTCCTCAC |
| G6Pase | CGACTCGCTATCTCCAAGTGA | GTTGAACCAGTCTCCGACCA |
| Pfkfb1 | ATGAGCTGCCCTATCTCAAGT | GTCCCGGTGTGTGTTCACAG |
| Gck | AGGAGGCCAGTGTAAAGATGT | CTCCCAGGTCTAAGGAGAGAAA |
| Pygl | GAGAAGCGACGGCAGATCAG | CTTGACCAGAGTGAAGTGCAG |
| Gys2  CTRP7 | ACCAAGGCCAAAACGACAG  GACGAGTCTTGCCATCTGTGC | GGGCTCACATTGTTCTACTTGA  TTAGCCTGATTGGCCCGAG |
| β-actin | CGTGCGTGACATCAAAGAGAAG | CAAGAAGGAAGGCTGGAAAAGA |

PEPCK, phosphoenolpyruvate carboxykinase; G6Pase, glucose-6-phosphatase; Pfkfb1, 6-phosphofructo-2-kinase/fructose-2, 6-biphosphatase1; Gck, glucokinase; Pygl, glycogen phosphorylase L; Gys2, glycogen synthase 2; β-actin, beta actin.

**Supplementary Table 2.** The correlation analysis of serum CTRP7 levels with other variables in study population.

|  | *Simple* | | *Multiple* | |
| --- | --- | --- | --- | --- |
| Variable | *r* | *p-*value | *β* | *p-*value |
| Age (yr) | 0.019 | 0.638 |  |  |
| BMI (kg/m^2^) | 0.414 | < 0.001 | 0.274 | < 0.001 |
| WHR | 0.271 | < 0.001 |  |  |
| FAT (%) | 0.132 | 0.001 |  |  |
| SBP (mmHg) | 0.236 | < 0.001 |  |  |
| DBP (mmHg) | 0.241 | < 0.001 |  |  |
| FBG (mmol/L) | 0.593 | < 0.001 |  |  |
| 2h-BG (mmol/L) | 0.610 | < 0.001 |  |  |
| HbA1c (%) | 0.563 | < 0.001 | 0.321 | < 0.001 |
| TG (mmol/L) | 0.336 | < 0.001 |  |  |
| TC (mmol/L) | 0.158 | < 0.001 |  |  |
| HDL-C (mmol/L) | -0.227 | < 0.001 |  |  |
| LDL-C (mmol/L) | 0.278 | < 0.001 |  |  |
| FFA (µmol/L) | 0.322 | < 0.001 |  |  |
| AUC_g_ | 0.604 | < 0.001 |  |  |
| HOMA-IR | 0.560 | < 0.001 | 0.191 | < 0.001 |
| HOMA-β | -0.384 | < 0.001 |  |  |
| Adiponectin (µg/mL) | -0.402 | < 0.001 | -0.143 | < 0.001 |

In multivariate linear regression analysis, values included for analysis were BMI, WHR, BP, FAT (%), HbA1c (%), FFA, TC, TG, HDL-C, LDL-C, HOMA-IR, Adiponectin.

| **Supplementary Table 3.** Association of circulating CTRP7 levels with IGT and nT2DM in fully adjusted models | |
| --- | --- |
| Model adjust | IGT T2DM |
|  | OR 95%CI *p-*value OR 95%CI *p-*value |
| Age, Sex | 1.017 1.010-1.023 < 0.001 1.046 1.039-1.053 < 0.001 |
| Age, Sex, BMI, WHR, FAT % | 1.015 1.008-1.021 < 0.001 1.047 1.039-1.055 < 0.001 |
| Age, Sex, BMI, WHR, FAT %, SBP, DBP | 1.015 1.008-1.022 < 0.001 1.046 1.038-1.055 < 0.001 |
| Age, Sex, BP, BMI, WHR, FAT %, lipid profile | 1.012 1.005-1.020 < 0.01 1.051 1.040-1.062 < 0.001 |

Results of binary logistic regression analysis are presented as the odds ratio (OR) of being in T2DM status increase in circulating. BMI, body mass index; WHR, waist-to-hip; FAT%, the percentage of fat *in vivo*; SBP, systolic blood pressure; DBP, diastolic blood pressure; lipid profile, including total cholesterol, FFA, triglyceride, LDL- and HDL-cholesterol.

**Supplementary Figure** **1.** ROC curve analyses were performed for the prediction of IGT **(A)** and T2DM **(B)**.

**Supplementary Figure 2.** **Lipid infusion induced insulin resistance *in vivo.*** **(A)** Glucose infusion rate (GIR). **(B)** M-value. Data are presented as mean ± SD. ***P*＜0.01 *vs.* Baseline.

**Supplementary Figure 3. Changes of serum CTRP7 level in cold-exposure test. (A)** Circulating CTRP7 levels in each individual before and after cold-exposure. **(B)** Average CTRP7 concentrations before and after cold-exposure in the study cohort. Data are presented as mean ± SD.

**Supplementary Figure 4. CTRP7 expression at mRNA and protein levels in Hepa1-6 cells.** Hepa1-6 cells were infected with pLVX-Puro or pLVX-*CTRP7* for 24h as indicated in the methods. **(A)** CTRP7 mRNA expression. **(B)** CTRP7 protein expression. Data are presented as mean ± SEM. ***P*＜0.01 *vs.* pLVX-Puro.
